# Stroke and dyslipidemia: clinical risk factors in the telestroke versus non-telestroke

**DOI:** 10.1186/s12944-018-0870-x

**Published:** 2018-09-27

**Authors:** Jordan Gainey, Brice Blum, Bekah Bowie, Keiko Cooley, Lee Madeline, Ervin Lowther Ervin, Thomas I. Nathaniel

**Affiliations:** 1 School of Medicine-Greenville, University of South Carolina, 607 Grove Road, Greenville, SC 29605 USA; 2 Greenville Health System, 701 Grove Road, Greenville, 29605 SC USA

**Keywords:** Acute ischemic stroke patients, Dyslipidemia, rtPA, Telestroke, Non-telestroke

## Abstract

**Background:**

Clinical risk factors related to not administering thrombolysis to acute ischemic stroke patients with incidence dyslipidemia is not clear. This issue was investigated in telestroke and non-telestroke settings.

**Methods:**

We analyzed retrospective data collected from a stroke registry to compare exclusion risk factors in the telestroke and non-telestroke. We performed multivariate analysis was performed to identify risk factors that may result in exclusion from rtPA. Variance inflation factors were used to examine multicollinearity and significant interactions between independent variables in the model, while Hosmer-Lemeshow test, Cox & Snell were used to determine the fitness of the regression models.

**Results:**

A greater number of patients with acute ischemic stroke with incidence dyslipidemia were treated in the non-telestroke (285) when compared with the telestroke network (187). Although non-telestroke admitted more patients than the telestroke, the telestroke treated more patients with rtPA (89.30%) and excluded less (10.70%), while the non-telestroke excluded from rtPA (61.40%). In the non-telestroke, age (adjusted OR, 0.965; 95% CI, 0.942–0.99), blood glucose level (adjusted OR, 0.995; 95% CI, 0.99–0.999), international normalized ratio (adjusted OR, 0.154; 95% CI, 0.031–0.78),congestive heart failure(CHF) (adjusted OR, 0.318; 95% CI, 0.109–0.928), previous stroke (adjusted OR, 0.405; 95% CI, 0.2–0.821) and renal insufficiency (adjusted OR, 0.179; 95% CI, 0.035–0.908) were all directly linked to exclusion from rtPA. In the telestroke, only body mass index (adjusted OR, 0.911; 95% CI, 0.832–0.997) significantly excluded acute ischemic stroke patients with incidence dyslipidemia from thrombolysis therapy.

**Conclusion:**

Despite having more patients with acute ischemic stroke that present incidence dyslipidemia, the non-telestroke patients had more clinical risk factors that excluded more patients from rtPA when compared with telestroke. Future studies should focus on how identified clinical risk factors can be managed to improve the use of rtPA in the non-telestroke setting. Moreover, the optimization of the risk-benefit ratio of rtPA by the telestroke technology can be advanced to the non-telestroke setting to improve the use of thrombolysis therapy.

## Background

The association between dyslipidemia and stroke is complicated and appears to vary depending on the stroke subtype, cholesterol levels, and lipid parameters [1]. For example, severe ischemic stroke and worse outcomes have been observed in patients that present with lower levels of cholesterol (TC) [2]. However, other studies [3, 4] found no association between levels of either TC or low-density lipoprotein (LDL-C), and stroke severity or outcome. In some studies, patients with lower total glyceride (TG) levels suffer more severe strokes [5, 6], while other studies showed higher TG levels in patients with a worse incidence stroke [7]. Contrarily, TG levels are not related to mortality and functional outcome [3, 8]. The conflicting results observed in dyslipidemia and ischemic stroke may be linked to the heterogeneity of stroke [9], and could affect the exclusion or inclusion of patients for recombinant tissue plasminogen (rtPA) irrespective of whether the patient is treated in the telestroke or non-telestroke setting.

A study comparing patients treated with rtPA in telestroke versus non-telestroke suggest that there is similarity in outcomes [10]; however, telestroke centers were shown to provide a twofold increase in the rates of rtPA [11]. Given the conflicting results of dyslipidemia’s effect on ischemic stroke, our first objective is to compare demographic and risk factors in patients with stroke that present with incidence dyslipidemia in the telestroke setting as compared with the non-telestroke. The presence or absence of specific risk factors in patients presenting with ischemic stroke and incidence dyslipidemia may contribute to the exclusion of patients from treatment with rtPA in either the telestroke or non-telestroke setting. One possibility is that acute ischemic stroke patients with incidence dyslipidemia are not present in the same proportions in the population of stroke patients in the telestroke and non-telestroke condition. For instance, since telestroke provided a twofold increase in the rates for rtPA, it is possible that more patients with acute ischemic stroke with incidence dyslipidemia may not receive thrombolytic therapy in the non-telestroke when compared with the telestroke. Therefore, the second objective investigated whether acute ischemic stroke patients that presented with a history of dyslipidemia are more susceptible to exclusion from rtPA in non-telestroke when compared with telestroke. Understanding the criteria for exclusion from rtPA in ischemic stroke patients with incidence dyslipidemia in the telestroke when compared with non-telestroke could provide an insight into the measurable risks that may be targeted to increase the usage of rtPA. Additionally, it could aid in identifying other potential research areas to increase eligibility for rtPA in the telestroke or non-telestroke network.

## Methods

### Patient selection

We collected retrospective data for consecutive rtPA-treated acute ischemic stroke patients from a regional stroke registry to study the exclusion criteria in patients presenting with acute ischemic strokes with incidence dyslipidemia in a telestroke versus non-telestroke study design. We have described the registry in previous studies [12–14]. Briefly, eligibility for rtPA was based on the AHA inclusion guidelines for the management of patients with acute ischemic stroke [15], and all cases of stroke were confirmed by computed tomography (CT). This study was approved by the institutional Committee for Ethics and the Institutional Review Board of the Greenville Health System. Patient information was retrieved from a database, and those with final discharge diagnoses inconsistent with ischemic stroke or transient ischemic attack were excluded. We collected two years data (2014 and 2016) from patients in the telestroke and non-telestroke. Baseline characteristics collected included NIHSS score and pre-rtPA systolic/diastolic blood pressures. A history of comorbid risk factors, including hypertension, diabetes mellitus, a previous stroke or transient ischemic attack, and `atrial fibrillation was also collected. Other comorbidities included carotid artery stenosis, hypertension, prosthetic heart valve, renal insufficiency, smoking, sleep apnea, migraine, obesity, and peripheral vascular disease. Demographic data were also collected including age, sex, race, and ethnicity. The study excluded patients that received endovascular therapy in order to maintain the homogeneity of the data. Acute ischemic stroke patients with incidence dyslipidemia without medical records were excluded as well. Both groups of patients were treated within similar time frames and the telemedicine evaluations were performed by stroke neurologists at the hub hospital. In the first 24 h, consultations for stroke were available to the spoke hospital and local neurologists were utilized to provide follow-up care. A stroke nurse collected data from patients that presented with signs and symptoms that could indicate acute ischemic stroke with incidence dyslipidemia. To confirm the presence or absence of a stroke, imaging results were utilized and then symptoms indices were used to correlate the results. In our data analysis, we defined dyslipidemia using the description of the American Heart Association that are consistent with the 95th percentile in the population when total cholesterol is greater than 5.2 mmol/L (200 mg/dl), LDL greater than 3.4 mmol/L (130 mg/dl), HDL less than 0.9 mmol/L (35 mg/dl), or triglycerides greater than 1.7 mmol/L (150 mg/dl) or current treatments that involve using a drug to lower cholesterol [16].

### Data analysis

We performed the initial exploratory data analyses using box plots, outlier estimation, and stem and leaf plots to discover extreme results, and missing variables were reviewed before analyses. All data were analyzed using the SPSS Statistics Software version 22.0 (Chicago, IL) and the significant level was determined at *P* < 0.05 for group comparisons. The patient data was de-identified and divided into the rtPA group and the no-rtPA group based on whether they received rtPA or not. The patient demographics, clinical variables, and comorbidities in the rtPA and no rtPA groups were compared by using two-tailed independent samples, and Student’s t-tests was considered for continuous variables. In addition, analysis were done to determine the mean, standard deviation, and range. For the categorical variables, Pearson’s Chi-Squared analysis was used, and the overall number of patients and percentage of patients for each variable was calculated. A regression analysis using Bayesian shrinkage was used to eliminate bias in variable selection and a relatively large number of potential covariates. The shrinkage enables reduced bias and stabilization of the model. To model the relationship between continuous variables and outcome, 25% of the total sample population was randomly selected and fractional polynomials were used to identify variables that provided the best fit in linearity. We retained variables if the resulting *p*-value was less than 0.001, and we excluded and eliminated stepwise all variables with *p*-values > 0.001. We re-added all previously eliminated variables and retained them in the model if the *p* < 0.001 criteria were fulfilled. For the final model, predicted parameters were estimated using standard errors and odds ratios. Additionally, all variables were calculated with a 95% asymptotic confidence interval. Variance inflation factors were utilized to evaluate multicollinearity and significant interactions between the independent variables.

## Results

In this study, 285 acute stroke patients with incidence dyslipidemia were admitted in the non-telestroke network, while 187 were admitted in the telestroke network (Table 1). Of the 285 patients in the non-telestroke, 110 received rtPA and 175 did not receive rtPA. Of the 187 patients treated in the telestroke, 167 received rtPA and 20 did not.

**Table 1:** Medical history, Clinical characteristics, and presenting symptoms of patients with acute ischemic stroke with a history of dyslipidemia. Continuous variables are represented as Mean ± S.D., and a Student’s t-test is used to compare between groups. Count (Percent Frequency) is used to represent discrete variables, and Pearson’s Chi-Squared is used to make comparisons between groups

Characteristic	Non-Telestroke (*N* = 285)	Telestroke (*N* = 187)	*P*-Value
Patient Age in Years			
Mean ± SD	70.5 ± 12.9	66.8 ± 12.9	0.002^*^
Age Group: No. (%)			
< 50 years	21 (7.4)	24 (12.8)	0.015^*^
50–59	37 (13)	24 (12.8)	
60–69	65 (22.8)	59 (31.6)	
70–79	85 (29.8)	48 (25.7)	
≥ 80	77 (27)	32 (17.1)	
Gender: No. (%)			
Male	138 (48.4)	101 (54)	0.235
Female	147 (51.6)	86 (46)	
Race: No. (%)			
Caucasian	232 (81.4)	148 (79.1)	0.056
African-American	29 (10.2)	31 (16.6)	
Other	2 (0.7)	3 (1.6)	
Hispanic Ethnicity: No. (%)	(0)	(0)	
Body Mass Index			
Mean ± SD	28.2 ± 6.6	31.2 ± 7.9	< 0.001^*^
Medical History: No. (%)			
Atrial Fib/Flutter	76 (26.7)	19 (10.2)	< 0.001^*^
Carotid Artery Stenosis	25 (8.8)	14 (7.5)	0.62
Congestive Heart Failure	38 (13.3)	21 (11.2)	0.499
Coronary Artery Disease	127 (44.6)	83 (44.4)	0.970
Depression	1 (0.4)	36 (19.3)	< 0.001^*^
Diabetes	124 (43.5)	92 (49.2)	0.225
Family History of Stroke	26 (9.1)	23 (12.3)	0.268
Hormone Replacement Therapy	3 (1.1)	5 (2.7)	0.182
Hypertension	259 (90.9)	168 (89.8)	0.707
Migraine	5 (1.8)	7 (3.7)	0.179
Obesity	104 (36.5)	111 (59.4)	< 0.001^*^
Peripheral Vascular Disease	29 (10.2)	19 (10.2)	0.996
Previous Stroke	103 (36.1)	50 (26.7)	0.033^*^
Previous TIA	51 (17.9)	26 (13.9)	0.251
Prosthetic Heart Valve	6 (2.1)	0 (0)	0.046^*^
Renal Insufficiency	20 (7)	14 (7.5)	0.847
Sleep Apnea	0 (0)	11 (5.9)	< 0.001^*^
Smoking	75 (26.3)	40 (21.4)	0.223
Substance Abuse	11 (3.9)	2 (1.1)	0.07
Initial NIH Stroke Scale			
Mean ± SD	10.4 ± 8.7	8.8 ± 7.7	0.05
Initial Labs & Vitals			
Total Cholesterol	161.7 ± 48.6	160 ± 40.3	0.387
Triglycerides	135.4 ± 85.6	142.6 ± 87.3	0.525
HDL	40 ± 13.4	39.2 ± 11.8	0.855
LDL	95.4 ± 37.2	96 ± 33.6	0.434
Lipids	6.6 ± 1.8	6.4 ± 1.6	0.024^*^
Blood Glucose	152.4 ± 88.7	135.2 ± 65.1	0.032^*^
Creatinine	1.3 ± 0.9	1.1 ± 0.6	0.002^*^
INR	1.2 ± 0.5	1 ± 0.2	0.004^*^
Heart Rate	81.3 ± 18	76.9 ± 15.1	0.061
Systolic Blood Pressure	151.1 ± 29.9	146.5 ± 23.7	0.333
Diastolic Blood Pressure	81 ± 18.8	79.4 ± 16	< 0.001^*^
Medications Prior to Admission: No. (%)			
Antiplatelet or Anticoagulant	195 (68.4)	122 (65.2)	0.472
Antihypertensive	237 (83.2)	152 (81.3)	0.601
Cholesterol Reducer	211 (74)	142 (75.9)	0.642
Diabetic Medication	91 (31.9)	70 (37.4)	0.217
Ambulation Status Prior to Event: No. (%)			
Ambulate Independently	247 (86.7)	176 (94.1)	0.065
Ambulate With Assistance	15 (5.3)	3 (1.6)	
Unable to Ambulate	13 (4.6)	4 (2.1)	
Not Documented	10 (3.5)	4 (2.1)	
Ambulation Status on Admission: No. (%)			
Ambulate Independently	41 (14.4)	41 (21.9)	0.019^*^
Ambulate With Assistance	48 (16.8)	43 (23)	
Unable to Ambulate	94 (33)	44 (23.5)	
Not Documented	102 (35.8)	59 (31.6)	
Ambulation Status on Discharge: No. (%)			
Ambulate Independently	120 (42.1)	107 (57.2)	0.009^*^
Ambulate With Assistance	86 (30.2)	48 (25.7)	
Unable to Ambulate	56 (19.6)	22 (11.8)	
Not Documented	23 (8.1)	10 (5.3)	
First Care Received: No. (%)			
Emergency Department	259 (90.9)	48 (25.7)	< 0.001^*^
Direct Admission	26 (9.1)	139 (74.3)	
rtPA Administration	110 (38.6)	167 (89.3)	< 0.001^*^
Improved Ambulation	180 (63.2)	136 (72.7)	0.031^*^

Table 2 presents the clinical characteristics that are associated with rtPA status for the telestroke and non-telestroke acute ischemic stroke patients that have a history of dyslipidemia. As shown in the table, 175 dyslipidemia stroke patients in the non-telestroke and 20 patients in the telestroke were excluded from rtPA. Dyslipidemia stroke patients excluded from receiving rtPA in the non-telestroke were older (71.8 yr. ± 12.6 yr. vs. 68.5 yr. ± 13.2 yr), ambulate independently (17.7% vs 5.4%), and had a history of a previous stroke (41.4% vs 28.2%), renal insufficiency (9.7% vs 2.7%), high blood glucose (162.1 ± 101.5 vs 136.8 ± 60.4), high creatinine (1.4 ± 1.1 vs 1.2 ± 0.6), high INR (1.2 ± 0.7vs 1.1 ± 0.1), and low diastolic blood pressure (78.8 ± 19.1 vs 84.4 ± 17.9). For the telestroke, excluded patients were not likely to be using cholesterol reducers (11.4% vs 73.7%), ambulate independently (5.4% vs 19.2%), and were admitted to the ED (5.4% vs 23.4%).

**Table 2:** Medical history, clinical characteristics, and presenting symptoms of patients with acute ischemic stroke stratified by telestroke and rtPA status. Continuous variables are represented as Mean ± S.D., and a Student’s t-test is used to compare between groups. Count (Percent Frequency) is used to represent discrete variables, and Pearson’s Chi-Squared is used to make comparisons between groups

	Non-Telestroke			Telestroke		
	No rtPA (*N* = 175)	rtPA (*N* = 110)	*P*-Value	No rtPA (*N* = 20)	rtPA (*N* = 167)	*P*-Value
Characteristic						
Patient Age in Years						
Mean ± SD	71.8 ± 12.6	68.5 ± 13.2	0.035^*^	67.1 ± 12	66.7 ± 13	0.905
Age Group: No. (%)						
< 50 years	14 (8)	7 (6.4)	0.003^*^	1 (0.6)	23 (13.8)	0.715
50–59	17 (9.7)	20 (18.2)		4 (2.4)	20 (12)	
60–69	30 (17.1)	35 (31.8)		7 (4.2)	52 (31.1)	
70–79	62 (35.4)	23 (20.9)		5 (3)	43 (25.7)	
≥ 80	52 (29.7)	25 (22.7)		3 (1.8)	29 (17.4)	
Gender: No. (%)						
Male	79 (45.1)	59 (53.6)	0.162	13 (7.8)	88 (52.7)	0.297
Female	96 (54.9)	51 (46.4)		7 (4.2)	79 (47.3)	
Race: No. (%)						
Caucasian	140 (80)	92 (83.6)	0.667	16 (9.6)	132 (79)	0.328
African-American	19 (10.9)	10 (9.1)		2 (1.2)	29 (17.4)	
Other	2 (1.1)	0 (0)		1 (0.6)	2 (1.2)	
Hispanic Ethnicity: No. (%)	1 (0.6)	2 (1.8)	0.315	0 (0)	6 (3.6)	0.389
Body Mass Index						
Mean ± SD	27.9 ± 6.2	28.7 ± 7.2	0.369	32.4 ± 8.3	31.1 ± 7.9	0.498
Medical History: No. (%)						
Atrial Fib/Flutter	53 (30.3)	23 (20.9)	0.081	2 (1.2)	17 (10.2)	0.98
Carotid Artery Stenosis	19 (10.9)	6 (5.5)	0.117	0 (0)	14 (8.4)	0.178
Congestive Heart Failure	29 (16.6)	9 (8.2)	0.043	2 (1.2)	19 (11.4)	0.854
Coronary Artery Disease	77 (44)	50 (45.5)	0.81	9 (5.4)	74 (44.3)	0.953
Depression	1 (0.6)	0 (0)	0.427	4 (2.4)	32 (19.2)	0.928
Diabetes	76 (43.4)	48 (43.6)	0.973	13 (7.8)	79 (47.3)	0.135
Family History of Stroke	15 (8.6)	11 (10)	0.683	0 (0)	23 (13.8)	0.076
Hormone Replacement Therapy	2 (1.1)	1 (0.9)	0.851	0 (0)	5 (3)	0.433
Hypertension	160 (91.4)	99 (90)	0.683	16 (9.6)	152 (91)	0.123
Migraine	1 (0.6)	4 (3.6)	0.055	1 (0.6)	6 (3.6)	0.754
Obesity	64 (36.6)	40 (36.4)	0.972	11 (6.6)	100 (59.9)	0.675
Peripheral Vascular Disease	16 (9.1)	13 (11.8)	0.467	0 (0)	19 (11.4)	0.112
Previous Stroke	72 (41.1)	31 (28.2)	0.027^*^	3 (1.8)	47 (28.1)	0.209
Previous TIA	28 (16)	23 (20.9)	0.293	4 (2.4)	22 (13.2)	0.404
Prosthetic Heart Valve	6 (3.4)	0 (0)	0.05	0 (0)	0 (0)	N/A
Renal Insufficiency	17 (9.7)	3 (2.7)	0.025^*^	1 (0.6)	13 (7.8)	0.655
Sleep Apnea	0 (0)	0 (0)	N/A	1 (0.6)	10 (6)	0.859
Smoking	40 (22.9)	35 (31.8)	0.094	4 (2.4)	36 (21.6)	0.873
Substance Abuse			0.877	0 (0)	2 (1.2)	0.623
Initial NIH Stroke Scale						
Mean ± SD	9.8 ± 9.4	11.1 ± 7.8	0.247	6.6 ± 5.6	9 ± 7.9	0.255
Initial Labs & Vitals						
Total Cholesterol	163.6 ± 51.5	158.8 ± 44.1	0.441	152.1 ± 34.9	160.8 ± 40.8	0.386
Triglycerides	132.3 ± 82.2	139.9 ± 90.5	0.492	127.1 ± 59.4	144.4 ± 89.8	0.426
HDL	40.7 ± 15.1	38.9 ± 10.5	0.254	39.1 ± 8.8	39.2 ± 12.1	0.982
LDL	96.5 ± 36.9	93.7 ± 37.7	0.55	89.8 ± 28.2	96.7 ± 34.1	0.407
Lipids	6.7 ± 2	6.4 ± 1.6	0.29	6.9 ± 1.9	6.4 ± 1.6	0.196
Blood Glucose	162.1 ± 101.5	136.8 ± 60.4	0.009^*^	155.6 ± 73.3	132.7 ± 63.8	0.138
Creatinine	1.4 ± 1.1	1.2 ± 0.6	0.009^*^	1.2 ± 1	1.1 ± 0.5	0.674
INR	1.2 ± 0.7	1.1 ± 0.1	0.004^*^	1.1 ± 0.4	1 ± 0.1	0.55
Heart Rate	82.8 ± 19.2	79 ± 15.9	0.08	71.9 ± 12.3	77.5 ± 15.3	0.115
Systolic Blood Pressure	148.7 ± 30.6	154.9 ± 28.6	0.092	149.7 ± 20.7	146.1 ± 24	0.518
Diastolic Blood Pressure	78.8 ± 19.1	84.4 ± 17.9	0.014^*^	76.4 ± 11.3	79.8 ± 16.4	0.376
Medications Prior to Admission: No. (%)						
Antiplatelet or Anticoagulant	119 (68)	76 (69.1)	0.847	13 (7.8)	109 (65.3)	0.981
Antihypertensive	144 (82.3)	93 (84.5)	0.62	15 (9)	137 (82)	0.446
Cholesterol Reducer	129 (73.7)	82 (74.5)	0.876	19 (11.4)	123 (73.7)	0.035^*^
Diabetic Medication	56 (32)	35 (31.8)	0.974	8 (4.8)	62 (37.1)	0.802
Ambulation Status Prior to Event: No. (%)						
Ambulate Independently	142 (81.1)	105 (95.5)	0.007^*^	18 (10.8)	158 (94.6)	0.394
Ambulate With Assistance	13 (7.4)	2 (1.8)		1 (0.6)	2 (1.2)	
Unable to Ambulate	11 (6.3)	2 (1.8)		0 (0)	4 (2.4)	
Not Documented	9 (5.1)	1 (0.9)		1 (0.6)	3 (1.8)	
Ambulation Status on Admission: No. (%)						
Ambulate Independently	31 (17.7)	10 (9.1)	0.006^*^	9 (5.4)	32 (19.2)	0.045^*^
Ambulate With Assistance	37 (21.1)	11 (10)		3 (1.8)	40 (24)	
Unable to Ambulate	52 (29.7)	42 (38.2)		5 (3)	39 (23.4)	
Not Documented	55 (31.4)	47 (42.7)		3 (1.8)	56 (33.5)	
Ambulation Status on Discharge: No. (%)						
Ambulate Independently	70 (40)	50 (45.5)	0.748	13 (7.8)	94 (56.3)	0.533
Ambulate With Assistance	54 (30.9)	32 (29.1)		3 (1.8)	45 (26.9)	
Unable to Ambulate	35 (20)	21 (19.1)		2 (1.2)	20 (12)	
Not Documented	16 (9.1)	7 (6.4)		2 (1.2)	8 (4.8)	
First Care Received: No. (%)						
Emergency Department	160 (91.4)	99 (90)	0.683	9 (5.4)	39 (23.4)	0.036^*^
Direct Admission	15 (8.6)	11 (10)		11 (6.6)	128 (76.6)	
Improved Ambulation	102 (58.3)	78 (70.9)	0.031^*^	15 (9)	121 (72.5)	0.809

Data analysis using multivariate approach with Bayesian shrinkage (Table 3) showed that after adjustments for all risk factors and variables, obesity (adjusted OR, 3.059; 95% CI, 1.514–6.177), direct admission (adjusted OR, 30.346; 95% CI, 13.224–69.637) and rtPA administration (adjusted OR, 8.012; 95% CI, 3.26–19.687) were associated with the telestroke while systolic blood pressure (adjusted OR, 0.986; 95% CI, 0.973–0.999) was associated non-telestroke.

**Table 3:** The regression model to identify clinical risk factors in acute ischemic stroke population in the telestroke and non telestroke. Positive B values (Adjusted, OR > 1) represent the variables that are more closely related to telestroke patients while negative B values (Adjusted, OR < 1) represent the variables more closely related to non-telestroke patients. Interactions among independent variables and multicollinearity were analyzed. The model fitness was evaluated by applying the Hosmer-Lemeshow test (*p* = 0.031), Cox & Snell (R^2^ = 0.464), and classification table (overall correctly classified percentage = 87.1%)

Patients	B Value	Adj. Odds Ratio	Wald	*P* Value
Systolic Blood Pressure	−0.014	0.986 (0.973–0.999)	4.392	0.036^*^
Obesity	1.118	3.059 (1.514–6.177)	9.718	0.002^*^
Direct Admission	3.413	30.346 (13.224–69.637)	64.845	< 0.001^*^
rtPA Administration	2.081	8.012 (3.26–19.687)	20.579	< 0.001^*^
Constant	−1.486	0.226	1.922	0.166

Further adjusted analysis with binary logistical regression was carried out to determine risk variables related to exclusion or inclusion for rtPA in the stroke population as a whole (telestroke and non-telestroke; Table 4). Our analyses identified NIH Stroke Scale Score (adjusted OR, 1.048; 95% CI, 1.011–1.085) and telestroke (adjusted OR, 13.904; 95% CI, 6.417–30.129) to be associated with inclusion for rtPA, while increasing age (adjusted OR, 0.974; 95% CI, 0.953–0.995), blood glucose level (adjusted OR, 0.996; 95% CI, 0.992–1), INR (adjusted OR, 0.194; 95% CI, 0.051–0.733), and renal insufficiency (adjusted OR, 0.349; 95% CI, 0.118–1.033) were related to exclusion from rtPA.

**Table 4:** A stepwise regression model for exclusion or inclusion clinical risk factors in rtPA administration. Positive B values (Adjusted, OR > 1) represent the variables that are more closely related to rtPA administration while negative B values (Adjusted. OR < 1) represent the variables more closely related to rtPA exclusion. Interactions among independent variables and multicollinearity were analyzed. The model fitness was evaluated by applying the Hosmer-Lemeshow test (*P* = 0.305), Cox & Snell (R^2^ = 0.276), and classification table (overall correctly classified percentage = 77.3%)

Patients	B Value	Adj. Odds Ratio	Wald	*P* Value
Increasing Age	−0.026	0.974 (0.953–0.995)	5.607	0.018^*^
NIH Stroke Scale Score	0.047	1.048 (1.011–1.085)	6.704	0.01^*^
Blood Glucose at Presentation	−0.004	0.996 (0.992–1)	4.652	0.031^*^
INR	−1.639	0.194 (0.051–0.733)	5.852	0.016^*^
Renal Insufficiency	−1.053	0.349 (0.118–1.033)	3.617	0.057
Telestroke	2.632	13.904 (6.417–30.129)	44.511	< 0.001^*^
Constant	3.725	41.470	11.236	0.001^*^

In patients treated in non-telestroke (Table 5), age (adjusted OR, 0.965; 95% CI, 0.942–0.99), blood glucose level (adjusted OR, 0.995; 95% CI, 0.99–0.999), INR (adjusted OR, 0.154; 95% CI, 0.031–0.78), CHF (adjusted OR, 0.318; 95% CI, 0.109–0.928), a previous stroke event (adjusted OR, 0.405; 95% CI, 0.2–0.821), and renal insufficiency (adjusted OR, 0.179; 95% CI, 0.035–0.908) were related to exclusion from rtPA. On the other hand, antiplatelet or anticoagulant (adjusted OR, 2.632; 95% CI, 1.288–5.38) was associated with rtPA inclusion in the acute ischemic stroke patients with incidence dyslipidemia. In the telestroke (Table 6), only BMI (adjusted OR, 0.911; 95% CI, 0.832–0.997) was related to exclusion from rtPA in stroke patients with incidence dyslipidemia.

**Table 5:** A stepwise regression model for clinical factors in non-telestroke patients. Positive B values (Adjusted, OR > 1) represent the variables that are more closely related to rtPA administration while negative B values (Adjusted, OR < 1) represent the variables more closely related to rtPA exclusion. Interactions among independent variables and multicollinearity were analyzed. The model fitness was evaluated by applying the Hosmer-Lemeshow test (*P* = 0.287), Cox & Snell (R^2^ = 0.221), and classification table (overall correctly classified percentage = 65.5%)

Patients	B Value	Adj. Odds Ratio	Wald	*P* Value
Age	−0.035	0.965 (0.942–0.99)	7.754	0.005^*^
NIHSS	0.056	1.058 (1.016–.102)	7.408	0.006^*^
BGL	−0.005	0.995 (0.99–0.999)	4.665	0.031^*^
INR	−1.869	0.154 (0.031–0.78)	5.107	0.024^*^
CHF	−1.145	0.318 (0.109–.928)	4.393	0.036^*^
Previous Stroke	−0.904	0.405 (0.2–0.821)	6.294	0.012^*^
Renal Insufficiency	− 1.719	0.179 (0.035–.908)	4.311	0.038^*^
Anti-platelet medication	0.968	2.632 (1.288–5.38)	7.041	0.008^*^
Constant	4.547	94.316	11.372	0.001^*^

**Table 6:** A stepwise regression model explaining clinical factors and their strength of association with rtPA administration in telestroke patients. Positive B values (Adjusted, OR > 1) represent the variables that are more closely related to rtPA administration while negative B values (Adjusted, OR < 1) represent the variables more closely related to rtPA exclusion. Interactions among independent variables and multicollinearity were analyzed. The model fitness was evaluated by applying the Hosmer-Lemeshow test (*P* = 0.558), Cox & Snell (*R*^2^ = 0.035), and classification table (overall correctly classified percentage = 92.0%)

Patients	B Value	Adj. Odds Ratio	Wald	*P* Value
BMI	−0.093	0.911 (0.832–0.997)	4.104	0.043^*^
Constant	5.457	234.474	11.392	0.001^*^

## Discussion

Four major findings arose from this study. First, within the same time frame, more patients presenting with acute ischemic stroke with incidence dyslipidemia were admitted to the non-telestroke when compared with the telestroke. Second, although non-telestroke admitted more patients than the telestroke, more patients received rtPA and fewer patients were excluded from rtPA in the telestroke. Third, more clinical variables were related to rtPA inclusion in the telestroke when compared with the non-telestroke. Finally, more risk factors were related to exclusion from rtPA in the non-telestroke when compared with the telestroke in patients with acute ischemic stroke with incidence dyslipidemia. The primary outcome of this analysis was rtPA exclusion of patients with acute ischemic stroke with incidence dyslipidemia. Other outcomes included individual components of the ischemic stroke and comorbidities associated with stroke or dyslipidemia.

In the univariate analysis, triglycerides, total cholesterol, LDL-C, HDL-C, and lipids were not found to be significantly different in rtPA and non-rtPA groups for both the telestroke and non-telestroke settings. Lipid levels were higher in the non-telestroke than the telestroke. This effect was attenuated in the adjusted analysis indicating that the proportion of exclusion does not depend on the site of treatment, but on the stroke population with incidence dyslipidemia. We observed that elderly stroke patients with incidence dyslipidemia who present with a high blood glucose level, high INR, CHF, a previous incidence of stroke, and renal insufficiency were excluded from rtPA in the non-telestroke. High blood glucose [17], CHF [18, 19], high INR [20], and a prior stroke event in the last 3 months [21] are well documented risk factors for stroke that affect outcomes in thrombolysis therapy.

Although there are conflicting results on the effects that dyslipidemia has on acute ischemic stroke patients, our study reveals that elderly acute ischemic stroke patients with incidence dyslipidemia are associated with underlying risk factors, including high blood glucose levels, and are more likely to face exclusion from rtPA. Chronic hyperglycemia has been shown to be a major risk for stroke, and acute hyperglycemia related to a stroke is an indicator of poor patient diagnosis [22]. The immediate and robust control of chronic hyperglycemia may not provide added advantages and, at worst, could cause damage [23]. For this reason, a targeted and coordinated approach to diminish the risk of blood glucose in stroke patients with incidence dyslipidemia is important.

Our finding that patients with acute ischemic stroke with incidence dyslipidemia, who present with CHF are more likely to face rtPA exclusion is supported by reports that CHF is linked to an increased risk of thrombus formation and is also associated with an increased risk of stroke in a 3-fold [18, 24, 25]. In addition, stroke in CHF patients is linked to both higher mortality and poor treatment outcomes [26]. Therefore, it is possible that the complex interplay between CHF, dyslipidemia, and stroke may account for the observed risk posed by CHF during stroke [27]. The exclusion of patients with elevated INR, and patients presenting with a previous stroke event in the last 3 months in the non-telestroke setting is not surprising, as similar results have been proposed in previous studies [28–30]. It is possible that the combined effect of old age, coupled with elevated INR and a previous stroke event occurring in the last 3 months played a role in the exclusion criteria in patients with acute ischemic stroke with incidence dyslipidemia.

In general, more risk factors were linked to exclusion from rtPA in the non-telestroke when compared with the telestroke; because BMI was the only exclusion risk factor in the telestroke. When assessed individually, clinical risk factors that excluded patients in the non-telestroke group may not represent the likely predictors of risk of post-rtPA [31]. For this reason, identification of each clinical risk factor may not be an effective method to determine the patient eligibility for rtPA in either the telestroke or non-telestroke setting [32]. We observed that there are significant effects of clinical risk factors in excluding stroke patients with incidence dyslipidemia from rtPA in the non-telestroke group, whereas only BMI excluded patients from rtPA exclusion in the telestroke. The relationship between dyslipidemia and stroke is complicated and could affect the exclusion or inclusion of patients for rtPA irrespective of whether the patients are treated in the telestroke or non-telestroke setting. The fact that only BMI excluded patients from rtPA exclusion in the telestroke, suggests that the telestroke technology may optimize the risk-benefit ratio of rtPA to allow clinicians to accurately make good decisions in the treatment of acute ischemic stroke patients with incidence dyslipidemia.

There are several limitations in this study. An example of this is that data used in the study is retrospective, and suggest variability in the determination of confounding variables. Although we adjusted for confounding factors, our comparisons were focused on telestroke versus non-telestroke and not within the telestroke and non-telestroke groups. However, a strength of this study is its use of a large stroke center that monitors quality treatment in the telestroke and non-telestroke network. Because of this, our study is well equipped to evaluate both rtPA and exclusivity criteria in acute ischemic stroke patients with incidence dyslipidemia. Our study highlights the clinical significance of risk factors in elderly stroke patients (> 80) with incidence dyslipidemia, indicating that early identification of risk factors and treatment could improve outcomes with thrombolysis therapy.

## Conclusion

Although the role and interactive effects of individual risk factors in the exclusion from rtPA is not clear, the interaction between these factors and dyslipidemia suggests aggressive multiple risk-reduction measures for acute ischemic stroke patients with incidence dyslipidemia. This study contributes to existing literature in stroke and dyslipidemia by showing that the telestroke technology excluded fewer stroke patients with incidence dyslipidemia when compared with non telestroke, offering clues for thrombolysis therapy optimization in the treatment of acute ischemic stroke with incidence dyslipidemia.

## Abbreviations

Adjusted OR-: Adjusted odd ratio
BMI: Body mass index
CHF: Congestive heart failure
CI: Confidence interval
INR: International normalized ratio
LDL-C: Low-density lipoprotein
rtPA: Recombinant tissue plasminogen
TC: Total cholesterol
TG: Total glyceride
